# Crystal structures of the ligand-binding domain of human peroxisome proliferator-activated receptor δ in complexes with phenylpropanoic acid derivatives and a pyridine carboxylic acid derivative

**DOI:** 10.1107/S2053230X22000449

**Published:** 2022-01-31

**Authors:** Takuji Oyama, Kazuki Takiguchi, Hiroyuki Miyachi

**Affiliations:** a Department of Biotechnology, Faculty of Life and Environmental Sciences, University of Yamanashi, Japan; b Lead Exploration Unit, Drug Discovery Initiative, The University of Tokyo, Japan

**Keywords:** nuclear receptors, peroxisome proliferator-activated receptor δ, PPARδ, ligand-binding domain

## Abstract

Peroxisome proliferator-activated receptor δ (PPARδ) is a member of the nuclear receptor family and regulates glucose and lipid homeostasis in a ligand-dependent manner. Numerous phenylpropanoic acid derivatives targeting three PPAR subtypes (PPARα, PPARγ and PPARδ) have been developed towards the treatment of serious diseases such as lipid-metabolism disorders. In spite of the increasing attraction of PPARδ as a pharmaceutical target, only a limited number of protein–ligand complex structures are available. Here, four crystal structures of the ligand-binding domain of PPARδ in complexes with phenylpropanoic acid derivatives and a pyridine carboxylic acid derivative are described, including an updated, higher resolution version of a previous studied structure and three novel structures. These structures showed that the ligands were bound in the ligand-binding pocket of the receptor in a similar manner but with minor variations. The results could provide variable structural information for the further design and development of ligands targeting PPARδ.

## Abstract 2

Structures of the ligand-binding domain of human peroxisome proliferator-activated receptor δ in complexes with three novel ligands (JKPL38, JKP39 and JK122) were determined. In addition, the structure of a previously reported complex was updated to higher resolution by obtaining better quality crystals.

## 1. Introduction

**Figure 1:**
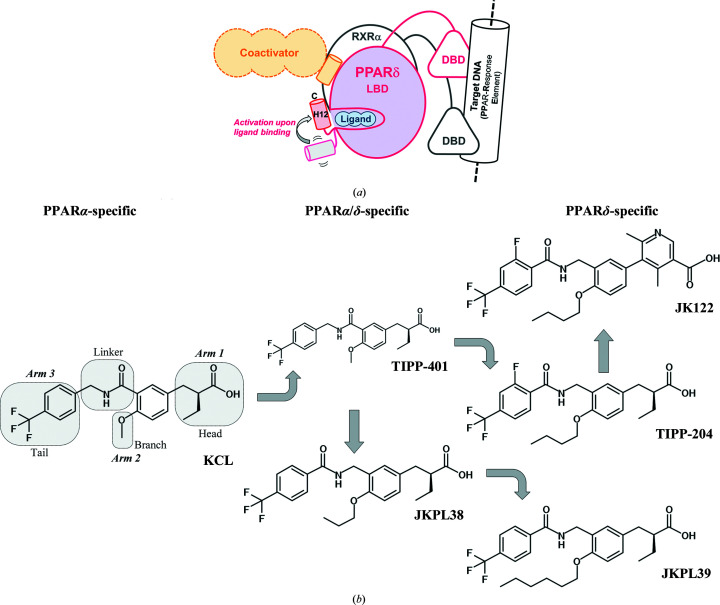
(*a*) A schematic diagram of the ligand-activated PPARδ–RXRα heterodimer. (*b*) Chemical structures of the ligands used in this study. Functional units are shown on the KCL structure with the binding arms in the ligand-binding pocket of PPARδ.

Nuclear receptors are transcription factors that are activated in a ligand-dependent manner (Georgiadi & Kersten, 2012 ▸; Dubois *et al.*, 2017 ▸). Among them, peroxisome proliferator-activated receptors (PPARs) consist of three subtypes: PPARα, PPARγ and PPARδ. They are involved in glucose and lipid homeostasis despite their different pharmacological properties and tissue distributions. Upon binding of agonists, PPARs adopt the activated conformation to interact with co-activator proteins, and then further bind the target DNA sequences as heterodimers with the retinoid X receptor (Fig. 1 ▸ *a*; Chandra *et al.*, 2008 ▸). PPARα and PPARγ have long attracted attention as targets for the development of therapeutic agents for hyperlipidemia and diabetes, respectively (Mirza *et al.*, 2019 ▸). PPARδ has not received as much attention as the other two subtypes, probably due to its wide distribution in the body. However, recent studies have indicated that PPARδ is involved in various diseases such as a variety of cancers (Müller, 2017 ▸), non-alcoholic fatty liver disease (NAFLD; Zarei *et al.*, 2021 ▸) and neuroinflammation (Strosznajder *et al.*, 2021 ▸). Therefore, PPARδ has recently gained attention as a target for therapeutic drug development. Since the first structural report of the ligand-binding domain of PPARδ (PPARδ LBD) in complex with intrinsic fatty acids and with a synthetic ligand (Xu *et al.*, 1999 ▸), numerous protein–ligand complex structures have been reported. Recently, Wu *et al.* (2017 ▸) reported 17 crystal structures of PPARδ LBD–ligand complexes. Nonetheless, the number of PPARδ LBD–ligand complex structures deposited in the Protein Data Bank (PDB) is still limited to 43 (as of 17 November 2021), which is fewer than those of PPARα (60) and PPARγ (254).

We have developed a series of phenylpropanoic acid derivative compounds targeting the three PPAR subtypes based on structure–activity relationship (SAR) studies, using the PPARα-specific compound KCL as a lead compound (Fig. 1 ▸ *b*; Miyachi, 2021 ▸). When we view the ligand-binding pockets of PPARs as Y-shaped with three arms (arms 1, 2 and 3), these compounds are bound so that the head, branch and tail portions fit into each arm (Oyama *et al.*, 2009 ▸, 2021 ▸). PPARδ-specific agonists possess a longer alkoxy residue (typically butoxy to *n*-hexyloxy) at the branch position, while PPARα/δ dual agonism is exhibited when the alkoxy part is replaced with a shorter part (methoxy to propoxy) (Kasuga *et al.*, 2007 ▸). We determined crystal structures of PPARδ LBD in complex with TIPP-401 (an α/δ dual agonist) and TIPP-204 (δ-specific; EC_50_ = 1.9 n*M*) (Oyama *et al.*, 2009 ▸), and further developed other derivatives such as JKPL38, which is an α/δ dual agonist with a propoxy residue (EC_50_ = 6.8 n*M*), and JKPL39, a δ-specific agonist (EC_50_ = 45 n*M*) which has a longer *n*-hexyloxy portion at the branch (Fig. 1 ▸ *b*; Kasuga, Ishida *et al.*, 2009 ▸).

We also developed biphenyl carboxylic acid derivatives as PPARδ partial agonists and antagonists (Kasuga, Oyama *et al.*, 2009 ▸). Furthermore, in an attempt to improve the water-solubility of these compounds, we obtained the water-soluble PPARδ-specific partial agonist JK122 (EC_50_ = 76 n*M*) with a dimethylpyridine carboxylic acid as the head group (Kasuga *et al.*, 2010 ▸). However, their binding mode to PPARδ LBD has not been examined experimentally, which has partly limited the SAR study cycle to obtain better ligands.

In this study, we have slightly modified the expression protocol of PPARδ LBD in *Escherichia coli* and thereby succeeded in the crystallization and structure determination of four protein–ligand complexes. We previously reported a crystal structure of PPARδ LBD–TIPP-204 at 3.0 Å resolution. Here, we obtained a higher resolution (1.9 Å) crystal of this complex, which updated the binding mode of TIPP-204. We also determined three novel structures of PPARδ LBD complexed with JKPL38, JKPL39 and JK122. These structures could provide useful information for the further development of better ligands for PPARδ.

## 2. Materials and methods

### 2.1. Macromolecule production

The protein expression (Table 1 ▸) and purification of human PPARδ LBD were performed using almost the same procedure as described previously (Oyama *et al.*, 2009 ▸), except for the use of a different *E. coli* expression cell strain: Arctic­Express (DE3) (Agilent Technology). This strain expresses two chaperone proteins derived from a microorganism in the Antarctic Ocean when exposed to a low temperature of around 286 K. The cells were transformed using the pET-28a vector, into which the PPARδ LBD gene was inserted, and were cultured in Terrific Broth at 310 K until the optical density (OD_600_) reached 0.6–0.8. The cells were rapidly cooled to 286 K using a bucket of ice water, and isopropyl β-d-1-thiogalactopyranoside (IPTG) was then added to the culture medium to a final concentration of 1 m*M* for the expression of PPARδ LBD. The cells were further incubated at 286 K for 48 h and harvested by centrifugation (3752*g*, 10 min at 277 K). Protein purification using three-step column chromatography (a nickel-chelating column followed by cation-exchange and gel-filtration columns) was conducted as described previously. The purified protein was concentrated to 7 mg ml^−1^ using Amicon Ultra concentrators (molecular-weight cutoff 3000). To form protein–ligand complexes, an aliquot of a concentrated solution of each ligand in 100% dimethyl sulfoxide (DMSO) was added to the protein solution at a protein:ligand molar ratio of 1:5 and was then used for crystallization.

### 2.2. Crystallization

Complexes of PPARδ LBD with TIPP-204, JKPL38 and JKPL39 were successfully crystallized by the hanging-drop vapor-diffusion method using the previously reported crystallization reservoir solution (reservoir *A* in Table 2 ▸; Oyama *et al.*, 2009 ▸). Plate-like crystals (with average dimensions of approximately 50 × 50 × 25 µm) were obtained within several days. The crystals were cryo-harvested using reservoir solution supplemented with 20%(*w*/*v*) PEG 1000 and flash-cooled in liquid nitrogen. For crystallization of the complex with JK122, crystallization screening was performed using a Mosquito semi-automatic dispensing robot (TTP Labtech) with the commercially available screening kits JCSG Core Suites I–IV (NeXtal). Diffraction-quality crystals were obtained by the hanging-drop vapor-diffusion method, in which the protein–ligand complex solution was mixed with a reservoir solution consisting of 0.2 *M* potassium thiocyanate, 20%(*w*/*v*) PEG 3350 (JCSG Core I Suite condition C9) in a 1:1 ratio (reservoir *B* in Table 2 ▸). Plate-like crystals similar to those of the abovementioned complexes were obtained within several days and were cryo-harvested using reservoir solution supplemented with 20%(*w*/*v*) glycerol.

### 2.3. Data collection and processing

X-ray diffraction data were collected on the AR-NE3A beamline at the Photon Factory (PF), Tsukuba, Japan. Diffraction images were recorded on an ADSC Quantum 270 CCD detector for the PPARδ LBD–JK122 crystals or a Dectris PILATUS 2M-F pixel-array detector for the other crystals. Data were processed with *XDS* (Kabsch, 2010 ▸) and *AIMLESS* (Evans & Murshudov, 2013 ▸). Statistics of X-ray diffraction data collection and processing are summarized in Table 3 ▸.

### 2.4. Structure solution and refinement

The crystal structures were solved by *Phaser* in the *Phenix* suite (Liebschner *et al.*, 2019 ▸) using a previously determined PPARδ LBD–ligand complex structure (PDB entry 2znq; Oyama *et al.*, 2009 ▸) as a probe. Structure refinement was then performed by iterations of manual model rebuilding in *Coot* (Emsley *et al.*, 2010 ▸) and crystallographic refinement using *phenix.refine* in *Phenix*. Statistics of structure refinement are summarized in Table 4 ▸.

## 3. Results and discussion

### 3.1. Updated structure of the PPARδ LBD–TIPP-204 complex

**Figure 2:**
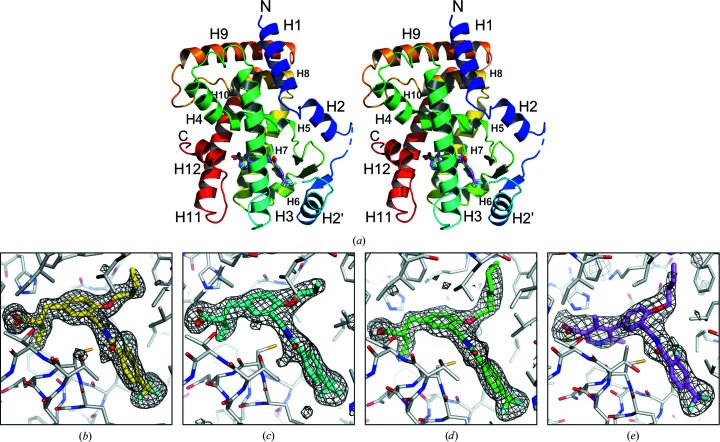
Structures of PPARδ LBD–ligand complexes. (*a*) Overall view of the complexes. PPARδ LDB is shown as a ribbon representation colored from blue (N-­terminus) to red (C-terminus). Omit *F*_o_ − *F*_c_ electron-density maps for (*b*) TIPP-204, (*c*) JKPL38, (*d*) JKPL39 and (*e*) JK122 are shown contoured at 3.0σ. Ligands are shown as stick models. C atoms of TIPP-204, JKPL38, JKPL39 and JK122 are colored yellow, cyan, green and violet, respectively. All atomic models were generated by *PyMOL* (http://www.pymol.org).

**Figure 3:**
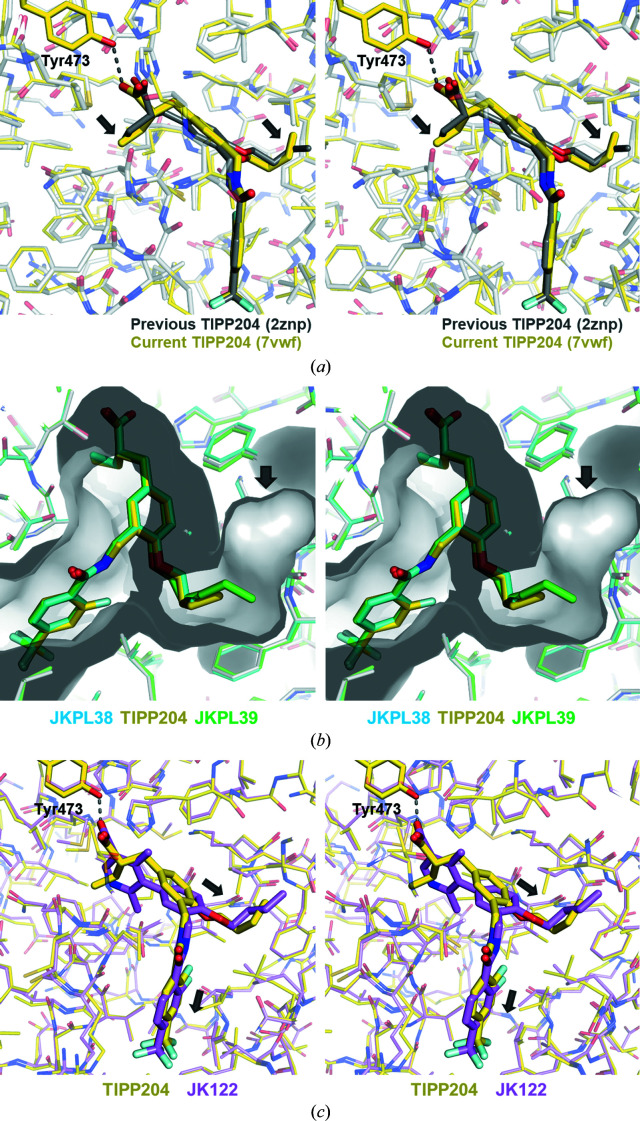
Ligand binding to PPARδ LBD. (*a*) Structure of PPARδ LBD–TIPP-204. A comparison is shown of the previous (gray) and current high-resolution (yellow) structures. Different conformations are indicated by arrows. (*b*) Superposition of PPARδ LBD in complexes with TIPP-204 (yellow), JKPL38 (cyan) and JKPL39 (green). The arm 2 groove is represented by a transparent surface. An arrow indicates the bottom of arm 2. (*c*) Structure comparison of PPARδ LBD–TIPP-204 (yellow) and PPARδ LBD–JK122 (violet). The branch butoxy residue and the tail trifluoromethylbenzyl residue shift slightly (about 0.8 Å) towards arms 2 and 3, respectively, compared with the corresponding parts of TIPP-204 (indicated by arrows).

As in our previous study, all of the crystals belonged to space group *P*2_1_, with two complex molecules in the asymmetric unit which are related by noncrystallographic twofold symmetry (Fig. 2 ▸ *a*). Both of the molecules in the asymmetric unit adopt the typical active conformation, in which Tyr473 in the C-terminal α-helix H12 generally forms a hydrogen bond to the carboxy group of the bound ligands in arm 1 to form the typical active conformation of H12, while in arms 2 and 3 the protein possesses hydrophobic pockets that interact with non­polar parts of the ligands (Figs. 2 ▸ *b*–2 ▸ *e*). We previously determined a 3.0 Å resolution structure of PPARδ LBD–TIPP-204 (PDB entry 2znp; Oyama *et al.*, 2009 ▸). In this study, the crystals of the same complex exhibited an improved resolution of 1.9 Å, which could be attributed to the modified protein-expression method, as protein purification and complex crystallization were performed under the same conditions. Indeed, the purification yield was increased to about 5 mg from a 1 l culture of *E. coli* cells, compared with around 1 mg in the previous protein-expression protocol. Comparison of the present (PDB entry 7vwe) and previous (PDB entry 2znp) structures of the ligand molecules in the ligand-binding pocket revealed only minor differences of the conformations of the alkyl groups, the head ethyl moiety and the branch butoxy residues, while the binding mode was unchanged overall (Fig. 3 ▸ *a*). The root-mean-square deviation (r.m.s.d.) for the corresponding 255 C^α^ atoms between the present and previous structures (calculated using the *SSM superpose* option of *Coot*) is 0.45 Å.

### 3.2. Structures of the PPARδ LBD–JKPL38 and PPARδ LBD–JKPL39 complexes

JKPL38, TIPP-204 and JKPL39 have successively longer *n*-alkoxy groups on their branches, which are accommodated in arm 2 of the ligand-binding pocket (Fig. 1 ▸). PPARδ has a deeper arm 2 pocket than PPARα. Our previous studies have shown that compounds with methoxy to propoxy groups tend to function as dual PPARα/δ agonists, while those with longer groups than butoxy function as PPARδ-specific agonists. The conformations of the alkoxy groups of JKPL38, TIPP-204 and JKPL39 in the PPARδ ligand-binding pocket were very similar to each other (Fig. 3 ▸ *b*). There is no significant structural difference in the PPARδ LBD structure (the r.m.s.d.s for 258 corresponding C^α^ atoms are below 0.24 Å). A visual inspection implies that PPARδ may bind ligands with an *n*-heptyloxy group at the branch.

### 3.3. Structure of the PPARδ LBD–JK122 complex

The crystals of the PPARδ–JK122 complex obtained using the same crystallization conditions as used for the other ligand complexes were of poor quality. We then searched for crystallization conditions and obtained high-quality crystals under another condition. The crystals belonged to the same space group as the other crystals and had highly similar unit-cell parameters. JK122 is a water-soluble PPARδ-specific partial agonist developed on the basis of a biphenylcarboxylic acid compound and has a dimethylpyridine carboxylic acid unit at the head (Fig. 1 ▸; Kasuga *et al.*, 2010 ▸). Consequently, the head of JK122 is slightly larger than the phenylpropaonic acid units of the other three ligands, and compared with them JK122 has one additional carbon in the connecting part between the head carboxy group and the linker benzene ring. Comparing the binding mode of JK122 in the ligand-binding pocket with that of TIPP-204 (the r.m.s.d. is 0.43 Å for the superposition of 251 corresponding C^α^ atoms), the head carboxy group forms a hydrogen bond to Tyr473 on H12 in a quite similar manner, including the location and direction. On the other hand, the rest of JK122 is pushed out towards arms 2 and 3 (by about 0.8 Å; Fig. 3 ▸ *c*). It is currently unclear whether this slightly different mode of binding accounts for the difference in function between JK122 (a partial agonist) and TIPP-204 and the other ligands (full agonists).

## 4. Concluding remarks

We determined four crystal structures of PPARδ LBD–ligand complexes, one of which is a high-resolution updated version, while the others are novel structures. A key to the success of this study was the increased stability of PPARδ LBD due to its co-expression with the low-temperature-induced chaperone proteins in the host cells. The three phenylpropanoic acid derivatives bound to the ligand-binding pocket in a similar conformation, despite the variation in the alkoxy chain length in the branch portion. This was accomplished by the wide arm 2 groove that is characteristic of PPARδ. JK122 was located similarly to other ligands in the ligand-binding pocket around the head region in spite of its larger size, while the rest of the ligand shifted slightly towards arms 2 and 3. The previously developed biphenyl carboxylic acid derivatives (Kasuga, Oyama *et al.*, 2009 ▸), which possess similar molecular structures to JK122, may bind to PPARδ LBD in a similar manner to JK122. The present structures are expected to provide useful structural information for the future design and synthesis of novel compounds with improved characteristics.

## Supplementary Material

PDB reference: PPARδ LBD–JK122, 7vwe

PDB reference: PPARδ LBD–TIPP-204, 7vwf

PDB reference: PPARδ LBD–JKPL38, 7vwg

PDB reference: PPARδ LBD–JKPL39, 7vwh

## Figures and Tables

**Table 1:** Macromolecule-production information

Source organism	*Homo sapiens*
Expression vector	pET-28a
Expression host	*Escherichia coli* BL21 (DE3)
Complete amino-acid sequence of the construct produced	GSHMPQVADLKAFSKHIYNAYLKNFNMTKKKARSILTGKASHTAPFVIHDIETLWQAEKGLVWKQLVNGLPPYKEISVHVFYRCQCTTVETVRELTEFAKSIPSFSSLFLNDQVTLLKYGVHEAIFAMLASIVNKDGLLVANGSGFVTREFLRSLRKPFSDIIEPKFEFAVKFNALELDDSDLALFIAAIILCGDRPGLMNVPRVEAIQDTILRALEFHLQANHPDAQYLFPKLLQKMADLRQLVTEHAQMMQRIKKTETETSLHPLLQEIYKDMY

**Table 2:** Crystallization

Method	Hanging-drop vapor diffusion
Plate type	VDX plate
Temperature (K)	293
Protein concentration (mg ml^−1^)	7
Buffer composition of protein solution	20 m*M* HEPES, 10 m*M* DTT, 500 m*M* ammonium acetate pH 7.5
Composition of reservoir solution	*A*: 11–14%(*w*/*v*) PEG 4000, 200 m*M* KCl, 40 m*M* bis-Tris methane, 6%(*v*/*v*) 1,3-propanediol, 0.5%(*w*/*v*) *n*-heptyl-β-D-glucopyranoside, 1 m*M* EDTA, 1 m*M* CaCl_2_.
	*B*: 0.2 *M* potassium thiocyanate, 20%(*w*/*v*) PEG 3350.
Volume and ratio of drop	1 µl protein and 1 µl reservoir solution
Volume of reservoir (µl)	500

**Table 3:** Data collection and processing Values in parentheses are for the outer shell.

Complex	PPARδ LBD–JK122	PPARδ LBD–TIPP-204	PPARδ LBD–JKPL38	PPARδ LBD–JKPL39
Diffraction source	AR-NE3A, PF	AR-NE3A, PF	AR-NE3A, PF	AR-NE3A, PF
Wavelength (Å)	1.00000	1.00000	1.00000	1.00000
Temperature (K)	100	100	100	100
Detector	ADSC Quantum 270	Dectris PILATUS 2M-F	Dectris PILATUS 2M-F	Dectris PILATUS 2M-F
Space group	*P*2_1_	*P*2_1_	*P*2_1_	*P*2_1_
*a*, *b*, *c* (Å)	39.12, 92.30, 96.09	39.56, 94.25, 96.31	39.50, 94.31, 96.25	39.59, 94.14, 96.18
α, β, γ (°)	90, 98.06, 90	90, 97.32, 90	90, 97.37, 90	90, 96.64, 90
Mosaicity (°)	0.21	0.11	0.15	0.16
Resolution range (Å)	50.0–3.00 (3.18–3.00)	50.0–1.90 (1.93–1.90)	50.0–2.25 (2.27–2.20)	50.0–2.10 (2.16–2.10)
Total No. of reflections	51094 (8195)	187262 (11806)	119135 (9950)	134437 (11149)
No. of unique reflections	13610 (2163)	55080 (3528)	35214 (3011)	40370 (3313)
Completeness (%)	99.9 (99.9)	99.8 (99.9)	99.1 (99.9)	98.7 (99.7)
Multiplicity	3.7 (3.7)	3.3 (3.3)	3.4 (3.3)	3.3 (3.4)
〈*I*/σ(*I*)〉	7.9 (2.0)	12.8 (1.9)	12.8 (3.1)	14.3 (3.0)
*R*_meas_	0.117 (0.561)	0.044 (0.529)	0.073 (0.511)	0.062 (0.513)
Overall *B* factor from Wilson plot (Å^2^)	51.0	28.1	28.8	33.1

**Table 4:** Structure refinement Values in parentheses are for the outer shell.

Complex	PPARδ LBD–JK122	PPARδ LBD–TIPP-204	PPARδ LBD–JKPL38	PPARδ LBD–JKPL39
Resolution range (Å)	47.6–3.00	42.6–1.90	42.6–2.20	42.6–2.10
Final *R*_cryst_	0.2294	0.2018	0.2412	0.2052
Final *R*_free_	0.2592	0.2181	0.2657	0.2395
No. of non-H atoms				
Protein	4136	4169	4161	4169
Ligand	112	104	100	106
Water	9	215	78	68
R.m.s. deviations				
Bond lengths (Å)	0.002	0.003	0.002	0.005
Angles (°)	0.50	0.63	0.53	0.72
Average *B* factors (Å^2^)				
Protein	48.67	37.15	40.80	40.24
Ligand	46.84	32.05	38.92	36.99
Water	33.30	40.34	36.72	37.61
Ramachandran plot				
Favored regions (%)	97.60	97.82	97.42	97.02
Additionally allowed (%)	2.40	2.18	2.58	2.98
Outliers (%)	0.00	0.00	0.00	0.00
PDB code	7vwe	7vwf	7vwg	7vwh
